# Group-based physical activity interventions for postpartum women with children aged 0–5 years old: a systematic review of randomized controlled trials

**DOI:** 10.1186/s12905-021-01581-1

**Published:** 2021-12-28

**Authors:** L. R. Peralta, W. G. Cotton, D. A. Dudley, L. L. Hardy, Z. Yager, I. Prichard

**Affiliations:** 1 School of Education and Social Work, Faculty of Education and Social Sciences, The University of Sydney, Sydney, NSW Australia; 2 Macquarie School of Education, Faculty of Arts, Macquarie University, Sydney, NSW Australia; 3 School of Human Movement and Nutrition Sciences, Faculty of Health Sciences, The University of Queensland, Brisbane, QLD Australia; 4 Prevention Research Collaboration, Sydney School of Public Health, The University of Sydney, Sydney, NSW Australia; 5 Institute for Health and Sport, Victoria University, Melbourne, 3011 Australia; 6 Health and Exercise Sciences, College of Nursing and Health Sciences, Flinders University, Adelaide, SA Australia

**Keywords:** Postpartum women, Physical activity, Systematic review, Group-based programs

## Abstract

**Background:**

It is estimated that less than one third of women (28%) worldwide, are not sufficiently active, and there is evidence indicating physical activity (PA) participation is lower during pregnancy and the postpartum period. Despite the importance of educating and encouraging postpartum women to engage in PA, existing systematic reviews have only focused on examining the impact of individually tailored PA interventions and on specific postpartum populations such as women who are inactive (i.e., do not meet PA recommendations) or women at risk of gestational diabetes mellitus or postnatal depression. This review aims to fill this gap by examining the impact of group-based PA interventions on postpartum women’s PA levels or other health behavior outcomes.

**Methods:**

A systematic literature search was conducted using four electronic databases (MEDLINE, CINAHL, EMBASE and PsychInfo) of published studies between 1st January 2000 and 31st October 2020. Studies were included if they targeted postpartum women with no current health conditions, had children aged 0–5 years, and engaged postpartum women in a group-based PA program that reported PA or other health behavior outcomes. Out of a total of 1091 articles that were initially identified, six were included.

**Results:**

Group-based PA interventions were moderately successful in changing or increasing postpartum women’s self-reported PA levels and psychological wellbeing in the first 2 years of their offspring’s life. Overall, group-based PA interventions were not successful in changing or increasing postpartum women’s objectively measured PA levels, but only one study objectively measured postpartum women’s PA levels. Narrative synthesis highlights the heterogeneity of the outcomes and methodologies used, and the low to medium risk of bias in the included studies.

**Conclusion:**

To strengthen the evidence-base for group-based PA programs with postpartum women there is an on-going need for more rigorous randomised controlled trials of appropriate length (at least 3 months in duration) with an adequate dose of group-based PA sessions per week (to meet PA guidelines), and that utilise objective measures of PA. In addition, future PA interventions for this population should include, at the very least, fidelity and process data to capture the characteristics or design features that appeal most to postpartum women.

**Supplementary Information:**

The online version contains supplementary material available at 10.1186/s12905-021-01581-1.

## Background

Pregnancy, childbirth, and the postpartum period can present many challenges to women’s engagement in health-related behaviors. One notable health behavior challenge is a decrease in mothers’ engagement in regular physical activity (PA) [1]. PA is one of the most beneficial activities for pregnant and postpartum women, helping to reduce fatigue [2], increase quality of life, mental acuity, psychological wellbeing and reduce postpartum depressive symptoms [3, 4]. Regular PA can also provide a small protective effect against the development of gestational diabetes [5] and decrease the risk of developing future chronic health conditions [6]. Regular engagement in moderate-intensity PA (at an intensity of walking pace or higher) for at least 20 to 30 min per day on most or all days of the week has been recommended during pregnancy and the postpartum period by the American College of Obstetricians and Gynecologists (ACOG) [7] and the World Health Organisation [8]. Similarly, the 2018 PA Guidelines for Americans and Australians recommend 150 to 300 min per week of moderate intensity PA during pregnancy and the postpartum period to reduce the risk of excessive gestational weight gain, gestational diabetes mellitus, and symptoms of postpartum depression [9, 10].

Internationally, less than one third of women (28%) are not sufficiently active, [11] and there is evidence indicating PA participation is lower during pregnancy and the postpartum period [12, 13]. Furthermore, women often do not return to their pre-pregnancy PA levels within the first few years postpartum [14]. For example, data from the Australian Longitudinal Study on Women’s Health showed a large reduction in PA levels in the 3 years following childbirth [15]. There are several reasons for this decline. First, despite the PA recommendations, the importance of resuming PA after childbirth is often not made clear to women, and many women need guidance on how to begin or resume PA [16]. A study of women at around 7 weeks postpartum found almost half reported the desire for more information about a safe return to PA [17]. Another study among pregnant women who planned to engage in PA after their child’s birth found only 15% reported their health care professional discussed with them the appropriate time to resume PA [18]. As such, appropriate authorities such as health professionals and health care practitioners should do more to help postpartum women participate in PA, particularly once the early postpartum support such as mother and baby information and support programs, have ceased [19]. The pregnancy and postnatal period can therefore be considered as a missed opportunity for beginning or resuming PA [20], as mothers who maintain or increase their PA levels from pre-pregnancy through to the postpartum period, experience better wellbeing outcomes compared with women who do not [21, 22].

Studies have reported a range of challenges and barriers that postpartum women face when wanting to begin or resume PA after childbirth. Two postpartum studies have suggested the importance of considering individual and environmental factors prior to the implementation of any PA program [23, 24]. Individual factors are those related to the mothers’ own circumstances, including income, the number of children under her care, working around baby feeding and nap times/routines, fatigue, physical conditions related to pregnancy and birth (e.g., pelvic pain) and not being able to make time for themselves [23–26]. Other individual factors include a lack of social support such as child-care, support from peers to co-participate in PA and negative family attitudes and beliefs about parenting [22]. One growing area of research exploring individual factors and postpartum women’s PA levels highlight body image, shame, and body (dis)functionality as barriers to returning to and continuing participation in PA after childbirth [27]. In addition to many individual barriers, postpartum women may also experience environmental barriers including access to public transport, recreational facilities with adequate child-care, postpartum exercise programs with appropriate progressions for a safe return to PA, neighbourhood safety concerns, and lack of access to an informative health system [22]. Postpartum women who report fewer individual and environmental barriers are more active and those who report high levels of self-efficacy, can generally overcome one or more of these barriers to be physically active in the postpartum period [23].

Given the importance of educating and encouraging postpartum women to engage in PA, and recent campaigns to encourage PA among postpartum women [7, 8], various interventions have been developed, and research has identified core components of intervention effectiveness [28]. These core components are cited as the essential characteristics of an intervention, while other characteristics can be modified to suit the contextual needs of participants and settings [28]. The Template for Intervention Description and Replication (TIDieR) framework has been used to identify which core components are necessary for postpartum lifestyle interventions, based on those interventions which have had the greatest effect [29]. The TIDieR framework shows there is often significant heterogeneity among subgroups, suggesting other factors could be associated with the effectiveness of these interventions. Similarly, other research also reports there is no clear consensus on whether individual or group PA interventions are more effective for postpartum women due to the diversity of the population and the complex environmental and individualistic factors that each postpartum woman faces and needs to overcome during this period [30, 31].

In general, group-based interventions are widely used to promote health and to support health-related behavior change, including PA participation. Systematic and realist reviews show group-based interventions can be effective in increasing PA among the general population, particularly if members of a group interact, identify as a unit, and express a degree of cohesiveness towards accomplishing goals [32, 33]. Furthermore, previous research [32] indicates that group-based interventions may be an effective way to increase PA among high-risk populations and those most in need of health behavior intervention. However, a recent realist review [33] reported that 48 of 52 group-based PA interventions were successful (i.e., showed a positive effect in PA outcomes), with group-based interventions reported to be effective for: (a) both males and females, (b) those who are healthy and those who live with chronic medical issues, (c) when one or multiple group-based strategies are employed, and (d) when conducted in a workplace or in the community at large [33].

As postpartum women are less likely to engage in PA behaviors due to competing demands (i.e., caring duties, domestic responsibilities) and numerous individual and environmental barriers, there is a body of research focusing on enablers [23, 24] that suggests that the social support mechanisms (i.e., interaction and communication with others who are experiencing the same phase of life) and personal support mechanisms (i.e., appropriate, progressive activities) embedded in group-based interventions may help postpartum women overcome such barriers. However, no research has focused on the important intervention components and the most effective combination of strategies to employ within group-based PA programs for postpartum women [33].

Currently, there is no systematic review that aims to assess the impact of group-based PA programs on postpartum women’s PA levels. Existing systematic reviews have shown that individually tailored exercise interventions appear to increase PA among postpartum women in the short-term [34–36], however most of the included interventions focused on specific populations such as women who are not active, obese women or women at risk of gestational diabetes mellitus or postnatal depression. These reviews have highlighted that individual or personal PA programs are associated with positive changes in PA and postnatal depression if participants are closely supervised, the program has frequent weekly sessions that allow for postpartum women to attain PA recommendations, and the PA program is supplemented by theoretical strategies (e.g., PA advice and counselling, goal setting and self-monitoring) [34–36]. Therefore, it is relevant and timely to review the literature systematically for group-based PA interventions only and the core components of these group-based programs for the general population of postpartum women. It is essential to build evidence of the effectiveness of such interventions and advance current knowledge to inform decision makers and researchers in clinical and public health practice. Therefore, this systematic review identifies, evaluates, and summarizes the findings of all relevant group-based PA studies to examine the effects on postpartum women’s PA levels or other health behavior outcomes.

## Methods

This systematic review was registered in the International Prospective Register of Systematic Reviews (PROSPERO; Registration no. CRD42020214276; available from https:// www.crd.york.ac.uk/prospero/) and conducted and reported following the Preferred Reporting Items for Systematic Reviews and Meta-Analyses (PRISMA) statement [37]. A systematic literature search was conducted using electronic databases (MEDLINE, CINAHL, EMBASE and PsychInfo) of published studies between 1st January 2000 to 31st October 2020 to capture interventions that are reflective of the modern pressures that postpartum women face. The search strategy included the use of terms in four broad categories: (i) participants; (ii) PA; (iii) settings; and (iv) design.

More specifically, the strategy included searching for the following terms in the title and abstract fields: (i) postpartum OR post-partum OR postnatal OR post-natal OR puerperium OR postpartal OR post-partal OR lactating OR lactation OR “nursing women” OR breastfeeding OR breastfeeding OR “after birth” OR “following pregnancy” OR postpregnancy OR “post pregnancy” OR “following childbirth” OR “after delivery” OR “post childbirth” AND (ii)“physic* activ*” OR exercis* OR fitness AND (iii) group* OR team OR leadership OR facilitat* AND (iv) “randomized controlled trial*” OR “Clinical Trial*” OR “randomised controlled trial*” OR RCT OR “random allocation” OR “randomly allocated” OR “allocated randomly”.

Reference lists of included studies were manually searched for additional articles. The complete search query for each database is in Additional file 1.

### Inclusion and exclusion criteria

Studies were included if they: (i) targeted postpartum women with children aged 0–5 years; (ii) targeted postpartum women with no current health conditions (e.g., Type 2 diabetes, postnatal depression); (iii) engaged postpartum women in a group-based PA program and this was a compulsory component (i.e., not pregnant women as participants in a group-based PA program and followed them up in the postpartum period); and (iv) reported PA measured objectively (e.g., accelerometer-based PA) or subjectively (e.g., self-reported PA and other health behaviors) or other health behavior outcomes (i.e., mental health and wellbeing outcomes, self-efficacy, sedentary behaviors).

### Study selection, data extraction and analysis

The lead author (LP) initially screened all articles, after duplicates were removed in Endnote, based on title and abstracts for primary inclusion (Stage One). Following the initial screening, the lead author screened full text articles based on the inclusion criteria (Stage Two). In cases where there was uncertainty of whether a full-text article should be included, a second reviewer (WC) assessed the article. If there was a discrepancy, consensus was reached between LP and WC by discussion.

Characteristics and results of studies were extracted by two of the authors (LP, DD). Due to the heterogeneity of the group-based PA interventions, the outcomes and measurements, a meta-analytic approach was not appropriate. As such, a narrative synthesis was conducted using a previously published methodological framework [38]. A preliminary synthesis, by study design and outcome, was produced, identifying trends within and between studies. Studies were also analysed based on intervention content, components, and measures.

The methodological quality of the individual studies was assessed using an assessment scale derived from van Sluijs and colleagues [39] described in Table 1. For each included article, two reviewers (LH and ZY) independently assessed whether the assessed item was present or absent. If an item was not described sufficiently in the published paper it was allocated a ‘no’ score. For each article, agreement between reviewers for each article was set a priori at 80% [39] (i.e., reviewers were required to agree that the items were either present or absent for eight of the ten items). Where there was discrepancy, a third reviewer (LP) independently assessed that item.

**Table 1:** Methodological quality assessment criterion

Criterion	Description
A	Key baseline characteristics are presented separately for treatment groups (age, and one relevant outcome) and for randomised controlled trials, positive if baseline outcomes were statistically tested and results of tests were provided
B	Randomisation procedure clearly and explicitly described and adequately carried out in randomized controlled trials (generation of allocation sequence, allocation concealment and implementation)
C	Validated measures of outcomes assessed (validation in same age group reported and/or cited)
D	Drop out reported and ≤ 20% for < 6-month follow-up or ≤ 30% for ≥ 6-month follow-up
E	Blinded outcome variable assessments
F	Outcomes assessed a minimum of 6-months after pre-test
G	Intention to treat analysis for outcome(s) (participants analysed in group they were originally allocated to, and participants not excluded from analyses because of non-compliance to treatment or because of some missing data)
H	Potential confounders accounted for in outcome analysis (e.g., baseline score, group/cluster, age)
I	Summary results for each group + treatment effect (difference between groups) + its precision (e.g., 95% confidence interval)
J	Power calculation reported, and the study was adequately powered to detect hypothesized relationships

Adapted from van Sluijs et al. [38]

The analyses reported in the articles reviewed were extracted and summarised along with each study grade using the adapted van Sluijs [39] criteria described above and in Table 1. A narrative synthesis was conducted to produce the results of the individual quantitative studies.

## Results

### Results of search and selection strategy

**Fig. 1:**
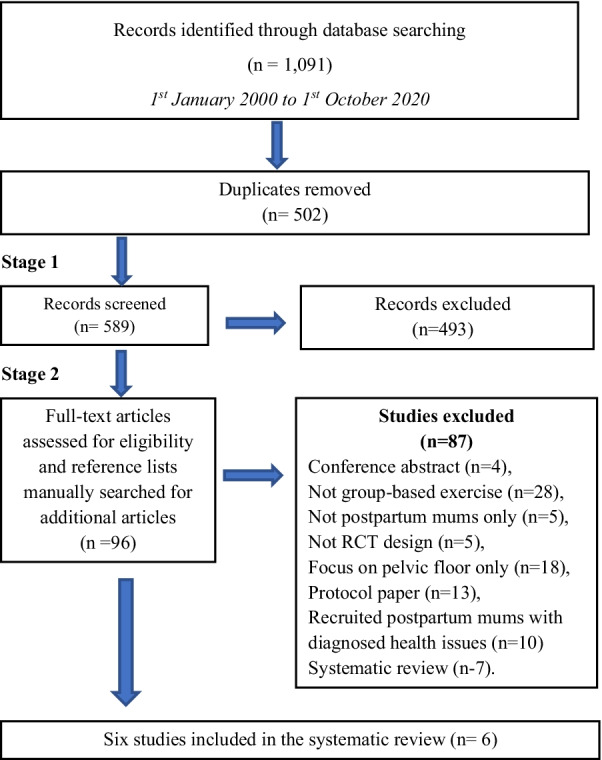
Flow diagram of studies included in the systematic review

The search retrieved 1091 peer-reviewed studies published in English between 1st January 2000 and 1st October 2020. After removing 502 duplicates, the titles and the abstracts of 589 studies were screened and 94 studies were considered for full-text review. A review of the full-texts and cross-referencing with existing systematic reviews in the field yielded six studies [with the MAMMiS study [40] having one peer-reviewed article and one PhD thesis [41]] for final inclusion in this systematic review and narrative synthesis. The most prevalent reasons for exclusion during full-text review were not a group-based PA program (e.g., had group activities like theory goal setting sessions, but PA sessions were conducted individually) and PA sessions only included pelvic strengthening activities (i.e., pelvic mobility exercises). A flow diagram of the study selection is show in Fig. 1.

### Description of studies

The characteristics of the studies meeting the inclusion criteria are summarised in Table 2. All six studies were published between 2006 and 2017 and were conducted in six different countries: Australia, Canada, Greece, Ireland, Scotland, and USA. The mean number of women participating in the intervention and control groups were 63 and 61, respectively (range for both groups was 16 to 225). In all six studies [40–46], women could be recruited as early as four to 6 weeks postpartum. Three of the studies [40, 42, 45] recruited women between 6 to 52 weeks postpartum. Therefore, in all the studies and throughout intervention duration, postpartum women participating in the group-based PA programs had at least one child between the ages from birth to 2 years. One study recruited first-time mothers only [45]. All the six studies were randomised controlled studies [40–46]. Four of the six studies designed and implemented group-based PA programs only [40, 42, 45, 46], with two of these studies also including behavior change techniques [40, 42]. Two studies utilised a multifaceted program which combined group-based PA sessions with either parental or nutrition education [43, 44]. One study embedded telephone support for the home component [42] and another integrated six telephone-counseling sessions lasting 20 min throughout the PA program [44], otherwise delivery in all other studies were face-to-face group-based sessions.

**Table 2:** An overview of the studies found in the systematic review and narrative analysis

Study	Design	Sample	Intervention (Type: Uni or multi-faceted, duration and intervention characteristics)	Outcomes
Cramp and Brawley [42] Canada	RCT	57 women who had given birth between 6 weeks and 1 year previously. Women were randomized to one of two conditions: Control: n = 31 standard exercise treatment (SE) Intervention: n = 26 standard exercise treatment plus group-mediated cognitive behavioral intervention (GMCB) Mean age: 31.5 years	Program: Uni-faceted with behavior change sessions Theoretical framework: Group-Mediated Cognitive Behavioral intervention (GMCB) Duration: 2 months (4 weeks supervised group training and 4 weeks home based phase) Group-based PA characteristics: Participants in both conditions participated in a 4-week intensive phase where participants received standard exercise training. Participants in the GMCB intervention group an additional 4 week home-based self-structured exercise. The first phase consisted of supervised, group delivered, centre-based standard exercise training fitness classes twice a week. Delivery of exercise to the group was through the same certified instructors at the same site. Intervention participants in the GMCB treatment received six, 20-min group-mediated cognitive behavioral training sessions on developing self-regulatory skills for self-management of PA and to overcome postpartum specific barriers to self-manage PA Comparison: Standard exercise treatment (SE) without the GMCB components Outcome measures: The 7-day Physical Activity Recall (7-PAR) questionnaire was utilized to assess self-reported PA and a 17-item modified version of Garcia and King's barrier efficacy scale measured self-regulatory efficacy	*At 2-month follow-up* Study retention was high (85%) and there was no selective effect of retention for either treatment Self-reported PA p < 0.01 Self-regulatory efficacy p < 0.05
Lee et al. [40] Scotland Gilinsky PhD thesis [41]	RCT	65 women who had given birth between 6 weeks and 1 year previously Intervention n = 33 Control n = 32 Mean age: 33.5 years	Program: “More Active MuMs in Stirling (MAMMiS)”; Uni-faceted, with behavior change sessions Theoretical framework: Transtheoretical Model (TTM) Duration: 3 months with 6 months follow-up Group-based PA characteristics: Participants could attend one session/week for 10 weeks and all women were encouraged to attend. Walks were conducted at a moderate-intensity (e.g., brisk pace) between 30 to 55 min per session Comparison: Traditional control group which received a NHS Health Scotland publication with information on physical activity guidelines and advice on implementation Outcome measures: postnatal weight, body composition, psychological general well-being Adapted General Well-Being Index (AGWBI), and fatigue using a visual analogue scale (VAS)	*At 6-month follow-up* Study retention was high, as all received the intervention or control condition and 91% (59/65) completed 6-month measurements Objective PA (counts p/min) p = 0.57 Postnatal weight (kg) p = 0.80 Body composition (BMI kg/m^2^) p = 0.80 Fat mass (kg) p = 0.81 Psychological well-being p = 0.19 Fatigue p < 0.01
Norman et al. [43] Australia	RCT	135 women recruited at time of hospital discharge Intervention n = 62 Control n = 73 Mean age: 29.3 years (intervention) 30.1 years (control)	Program: “Mother and Baby” (M&B); Multifaceted Theoretical framework: Not reported Duration: 2 months with 12-week follow-up Group-based PA characteristics: The intervention group received specialized exercise, 1 h per week, provided by a women’s health physical therapist combined with parenting education. Each week, women undertook one hour of group exercise with their babies, facilitated by a physical therapist, which involved cardiovascular and strength components. Each of the eight exercise sessions was adapted for each woman depending on the type of delivery and her recovery. Participants also had a 30-min parent education session delivered by health care professionals, including physical therapists, dietitians, speech pathologists, health psychologists, and midwives Comparison: The control group received a leaflet on parenting education mailed to them every week over 8 weeks Outcome measures: Psychological well-being (Positive Affect Balance Scale), depressive symptoms (Edinburgh Postnatal Depression Scale), and PA levels were assessed at baseline, after 8 weeks (post-program), and then 4 weeks later	*At 3-month follow-up* The retention rate for those commencing the study was 96% Psychological well-being p = 0.58 Depression p = 0.19 Self-reported PA (mins p/w) p = 0.87
Ostbye et al. [44] USA	RCT	450 women, enrolled 6 weeks postpartum, recruited through obstetrics clinics and community posters (stratified by black versus other and primiparous versus multiparous) using block randomization Intervention n = 225 Control n = 225 Mean age: 30.9 years	Program: “Active Mothers Postpartum” (ACTIVMOMS); Multifaceted Theoretical framework: Social Cognitive Theory, Stage of Readiness, and Motivation models Duration: 9 months Group-based PA characteristics: Eight healthy eating classes (Mom’s Time Out [MTO]), ten physical-activity group sessions (ACTIVMOMS classes) and six telephone counselling sessions. Given the intervention’s strong emphasis on walking, a sport stroller was provided to encourage walking for exercise outside of class and after the end of the intervention. The ACTIVMOMS classes encouraged walking and demonstrated activities designed to enhance recovery from pregnancy, including aerobics, strength and flexibility training, and pelvic-floor exercises. Using a front-facing baby carrier, mothers could exercise with their babies in a mom-and-tot format if desired. Every 6 weeks, women received one of six counselling sessions from a trained counsellor, lasting about 20 min each Comparison: The control group received biweekly newsletters with general tips for postpartum mothers. Women in both groups received monetary incentives on completion of each follow-up assessment Outcome measures: self-reported physical-activity recall and television time (7-day physical-activity recall [7-PAR], food-frequency questionnaire [FFQ] and weight)	*At 9-month follow-up* The retention rate for those commencing the study was 94% Daily caloric intake p = 0.37 Self-reported PA (Vigorous intensity mins p/w) p = 0.99 TV hours/day p = 0.08 Weight (kg) p = 0.25
Timlin and Simpson [45] Ireland	RCT	32 first time mothers were recruited from a Sure Start Community Centre if they had a baby aged between 6 weeks to 1-year-old Intervention n = 16 Control n = 16 Mean age: 28 years	Program: Dru Yoga; Unifaceted Theoretical framework: Not reported Duration: 1 month Characteristics: The participants in the intervention group attended for a one-hour Dru yoga session, once a week, for 4 weeks. The Dru yoga programme consisted of four parts and was designed to meet the needs of postpartum women. The intervention group were also offered a 20-min Dru yoga DVD to take home and practice at least twice a week. Participants were asked to keep a diary and write in it each week how many times they completed the DVD Comparison: The control group received no treatment but received the Dru Yoga DVD at the completion of the intervention Outcome measures: Physical Activity Readiness Questionnaire (PAR-Q), perceived stress (Perceived Stress Scale), mood, (Positive and Negative Affect Schedules [PANAS]) and coping (Brief Cope)	*At 1-month follow-up* The retention rate for those commencing the study was 100% PA readiness = Not reported Perceived stress p = 0.85 Mood p = 0.24 Coping p = 0.27
Zourladani et al. [46] Greece	RCT	40 primiparous women recruited at four to 6 weeks postpartum Intervention n = 20 Control n = 20 Mean age: 31.3 years	Program: Unifaceted Theoretical framework: Not reported Duration: 3 months Characteristics: The intervention group participated in a low impact exercise training programme, involving 50–60 min of aerobic, stretching and strengthening exercise, three days a week, for 12 weeks. It began with a ten minute warm-up, including low impact dance aerobics and stretching of the main muscle groups, followed by 20–25 min of low impact aerobics. A 15–20-min muscle strengthening programme followed, which consisted of exercises for the upper and lower back, abdominal muscles, and pelvic floor. It was delivered by a fitness specialist Comparison: The control group did not take part in any exercise programme Outcome measures: psychosocial well-being (Lederman Postpartum Self-Evaluation Questionnaire), weight, and infant feeding method	*At 3-month follow-up* The retention rate for those commencing the study was not reported Weight (kg) p = 0.67 Psychosocial wellbeing p < 0.05

The control conditions differed among the studies. One study provided the same group-based PA program for control participants, but without the added self-regulatory behavioral skills training that was designed for the intervention group [42]. Three studies provided control participants with education material either weekly [43], bi-weekly [44] or as a one-off [40]. One study provided control participants with a yoga instructional DVD after the intervention ceased [45], and participants in the remaining study maintained their usual activities [46].

Three of the six interventions utilised theoretical frameworks to increase PA behaviors throughout the intervention [40–42, 44]. The frameworks were different for each of the three studies: Group-Mediated Cognitive Behavioral counselling [42]; Transtheoretical model [40, 41]; and a combination of Social Cognitive Theory, Stage of Readiness, and Motivation models [44]. The study using Group-Mediated Cognitive Behavioral counselling embedded six, 20-min group-mediated cognitive behavioral training sessions for the intervention participants to develop self-regulatory skills for self-management of PA and to overcome postpartum specific barriers to self-manage PA [42]. The study that was underpinned by the Transtheoretical model employed a health psychologist with experience in motivational interviewing to implement behavior change, walk leader training, and to deliver a face-to-face PA consultation (approximately 45 min in length) at the start of a 10-week group pram walking program and a second consultation (approximately 25 min in length) at the end of the program [40, 41]. The study using the combination of Social Cognitive Theory, Stage of Readiness, and Motivation models designed and delivered eight healthy eating classes (Mom’s Time Out [MTO]), ten physical-activity group sessions (ACTIVMOMS classes) and six telephone counselling sessions. Intervention participants were also provided with a study notebook with exercises, recipes, and other intervention-related information, a pedometer, and a sport stroller to encourage walking for exercise outside of class and at the end of the intervention [44].

Five of the six interventions were less than 3 months in length [40–43, 45, 46]. One study embedded a 4-week group-based PA program [45], two studies were 2 months in length [42, 43], and another two studies were 3 months in length [40, 46]. The other study was 9 months in length [44]. It is important to note though, that two studies had follow-up measures beyond post-intervention. Norman and colleagues [43] had a 3-month follow-up measurement, and Lee and colleagues [40] had a 6-month follow-up measurement.

All studies included a group-based PA intervention, however only five of the six studies had PA as their primary outcome [40–45]. Three of the study interventions used self-reported measures of PA [42–44] and one measured PA readiness [45] although the PA readiness results were not reported in the paper. Only one study objectively measured PA with accelerometers, with the results only published in the PhD thesis, not the publication [40, 41]. Three of the six interventions included anthropometric measures as secondary outcomes (body mass index, body weight, and fat mass) [40, 44, 46]. One of the multifaceted interventions measured caloric intake and sedentary behavior [44]. Five of the six studies had psychological outcomes and measures [40, 41, 43–46]. Of the five studies, three measured psychological wellbeing [40, 43, 46], but used different scales. The scales used were the Adapted General Well-Being Index (AGWBI) [40], Positive Affect Balance Scale (PABS) [43] and Lederman Postpartum Self-Evaluation Questionnaire: Measures of Maternal adaptation [46]. Other psychological outcomes measured in the studies included fatigue [40], postnatal depression [43], perceived stress, mood and coping [45]. One study measured self-regulatory efficacy [42].

### Methodological quality of studies

Table 3 summarises the methodological quality of the included interventions, adapted from van Slujis et al. [39]. Three interventions met ≥ six assessment criteria, which demonstrates a low risk of bias [43–45], with two interventions meeting five of the criteria [40, 46], and one study meeting four of the criteria demonstrating a medium risk of bias [42].

**Table 3:** Methodological quality assessment

Study	Methodological quality assessment items										Criteria met (n)
	A	B	C	D	E	F	G	H	I	J	
Cramp and Brawley (2006) [42]	Y	N	Y	Y	N	N	N	N	Y	N	4
Lee et al. (2016) [40]	Y	N	Y	Y	N	Y	N	N	Y	N	5
Norman et al. (2010) [43]	Y	Y	Y	Y	N	N	Y	Y	Y	Y	8
Ostbye et al. (2009) [44]	Y	Y	Y	Y	N	N	Y	Y	Y	Y	8
Timlin et al. (2017) [45]	Y	Y	Y	Y	N	N	N	N	Y	Y	6
Zourladani et al. (2011) [46]	Y	Y	Y	N	N	N	N	N	Y	Y	5

A: Key baseline characteristics are presented separately for treatment groups (age, and one relevant outcome) and for randomised controlled trials, positive if baseline outcomes were statistically tested and results of tests were provided; B: Randomisation procedure clearly and explicitly described and adequately carried out in randomized controlled trials; C: Validated measures of outcomes assessed; D: Drop out reported and ≤ 20% for < 6-month follow-up or ≤ 30% for ≥ 6-month follow-up; E: Blinded outcome variable assessments; F: Outcomes assessed a minimum of 6 months after pre-test; G: Intention to treat analysis for outcome(s); H: Potential confounders accounted for in outcome analysis; I: Summary results for each group + treatment effect (difference between groups) + its precision (e.g., 95% confidence interval); J: Power calculation reported, and the study was adequately powered to detect hypothesized relationships

All interventions reported baseline sociodemographic and behavioral characteristics of the women in both intervention and control groups, described validated measures, and presented results for each group, including treatment effect and precision. Only one study conducted a post-test at 6 months [40], and only two interventions accounted for potential confounds and conducted intention to treat analyses [43, 44]. None of the studies conducted blinded assessments.

### Summary of intervention effect

#### Self-reported PA

Group-based PA interventions were somewhat successful in changing or increasing postpartum women’ self-reported PA levels in the first 2 years of their offspring’s life. One of the four studies that measured self-reported PA reported a statistically significant effect at the completion of the 2-month group-based PA intervention (p < 0.01) [42]. This group-based intervention consisted of a 4-week intensive phase where participants received group delivered, centre-based fitness classes twice a week for 4 weeks. Delivery of exercise to the group was through the same certified fitness instructors at the same site. The second phase was home-based self-structured exercise. It is important to note that there were no follow-up measures conducted to measure the longer-term impact of this study [42]. The other two studies that measured self-reported PA [43, 44] had no effect on postpartum women’s PA at the completion of the intervention (44: p = 0.87; 45: p = 0.99). Further, the group-based PA intervention which reported significant changes in self-reported PA [42], was also successful in improving postpartum women’s self-regulatory efficacy skills (p < 0.05) [42], but it was the only study in this review that had this as an outcome of their study and therefore integrated an appropriate theoretical framework and intervention program. Intervention participants received six, 20-min group-mediated cognitive behavioral training sessions on developing self-regulatory skills for self-management of PA and to overcome postpartum specific barriers, which was important for the second phase of the PA intervention.

#### Objective PA

Overall, group-based PA interventions were not successful in changing or increasing postpartum women’ objectively measured PA levels in the first 2 years of their offspring’s life. However, only one study objectively measured postpartum women’ PA levels after a group-based PA intervention (p = 0.57) [40].

#### Behavior and anthropometric outcomes

Group-based PA interventions were not successful in changing or increasing a range of postpartum women’s behavior outcomes. The six studies included many different behavioral outcomes, including PA readiness [45]; weight, BMI and fat mass [40, 44, 45]; psychological wellbeing [40, 43, 46]; self-regulatory efficacy [42]; fatigue [40]; stress, mood and coping [45]; and caloric intake and sedentary behavior [44]. Of these outcomes, one of the three studies that included a psychological wellbeing outcome and measurement, produced a statistically significant improvement in postpartum women’s psychological wellbeing after a 3-month group-based PA intervention (p < 0.05) [46]. The other two studies did not produce a statistically significant improvement in postpartum women’s psychological wellbeing (42: p = 0.19; 44: p = 0.58). The one and only studies that measured fatigue [40] and self-regulatory efficacy [42], positively affected postpartum women’s feelings of fatigue and efficacy capabilities after participation in a 3-month and 2-month group-based intervention, respectively. Interestingly, women’s feelings of fatigue were still evident at the 6-month follow-up measurement period [40] (p < 0.01). There were not statistically significant changes in all the other behaviour outcomes.

Of the three studies that were underpinned by a theoretical framework [40, 42, 44], one was successful at significantly improving postpartum women’s self-reported PA (p < 0.01) [42] and self-regulatory efficacy (p < 0.05) [42], and one other study was successful at significantly improving postpartum women’s feelings of fatigue (p < 0.01) [40]. These studies embedded a range of behavior change strategies, including counselling sessions to develop self-regulatory skills for self-management of PA and to overcome postpartum specific barriers to self-managed PA [42] and workbooks to structure goal-setting, planning, and self-monitoring plans around PA and given pedometers to monitor PA [40]. The other study [44], which used a theoretical framework and had the longest intervention duration of 9 months, reported no significant changes to postpartum women’s daily caloric intake, self-reported PA, TV hours/day or body weight.

The three studies that were unifaceted in their approach (i.e., only focused on designing a group-based PA program with or without behavior change strategies) were all successful in making a statistically significant change in postpartum women’s self-reported PA (p < 0.01) [42], self-regulatory efficacy (p < 0.05) [42], fatigue (p < 0.01) [40], and psychological wellbeing (p < 0.05) [46]. This is important to note, as this was the only element of the six studies that led to all the statistically significant changes captured in the studies included in this review.

### Narrative synthesis

This synthesis refers and extends the information provide in Table 2. The six studies extracted for this review used different activities to design group-based PA programs for postpartum women. Four of the studies focused on delivering cardiovascular and strengthening activities for a period ranging from 40–60 min a session [42–44, 46], which would be considered appropriate activities for providing postpartum women with the opportunity to engage in moderate-to-vigorous intensity physical activities that are health enhancing [9, 10]. The cardiovascular and strengthening activities sessions included pelvic floor strengthening activities, which shows that the intervention designers were developing programs to meet the needs of postpartum women as they returned to PA after giving birth. Of the four studies that embedded these activities, only one [42] reported statistically significant changes in self-reported PA, with one study not stating and measuring PA as one of the study outcomes [46]. However, this study did report a statistically significant improvement in postpartum women’s psychological wellbeing [46], suggesting that there is a relationship between dose of physical activity and effects on psychological wellbeing for postpartum women, or that there is a ceiling effect if postpartum women have high measures of psychological wellbeing at the beginning of group-based PA program [40, 43]. It still unclear whether psychological wellbeing outcomes are attributable to changes in PA or the addition of social support mechanisms in group-based PA programs.

It also should be noted that only one of the studies designed a group-based PA programs that was co-designed and delivered by a women’s health specialist trained specifically to ensure that pelvic floor muscles are switched on and could provide appropriate advice and feedback to postpartum women during the activities [43]. Two of the studies used trained fitness professionals to deliver the PA programs [42, 46] but it was not mentioned whether these fitness professionals had engaged in further education to gain women’s health accreditation to support their knowledge and capabilities in this area.

The other two studies focused on pram walking and yoga [40, 45]. These activities are considered light to moderate intensity physical activities [47] and could be deemed appropriate for easing postpartum women back into PA after birth. However, as the dose of these interventions was only minimal, that is once per week for 40–60 min in duration, this would be insufficient for providing postpartum women with the opportunity to meet the minimum PA guidelines and to gain health enhancing benefits [9, 10]. This was particularly highlighted in Lee and colleagues’ study [40] where PA was measured at 3- and 6-month follow-up with no statistically significant improvement in objectively measured PA found at either time point. Five of the six studies would be deemed insufficient in terms of providing opportunities for postpartum women to attain the minimum requirements stated in the PA guidelines [40, 41, 43–45]. The only study that provided a minimum of 150 min of moderate intensity PA each week in their intervention was Zourladani’s study [46]. However, PA levels of postpartum women were not an outcome of the study and therefore not measured. The only other study that came close to providing sufficient PA sessions per week, according to the PA guidelines, was Cramp and Brawley’s study [42], which was the only study in this review reporting statistically significant increases in self-reported PA. It provided postpartum women with two sessions per week for a duration of 60 min and managed to plan for a home transition program through its group-mediated cognitive behavioral training sessions.

## Discussion

The purpose of this systematic review was to examine the extent to which group-based PA interventions for postpartum women increase PA or other health behavior outcomes. Our search located only six group-based PA interventions for postpartum women and substantial heterogeneity in the methods used to measure PA. Overall, the group-based PA interventions captured in this systematic review were not successful in increasing postpartum women’s objectively measured PA levels in the first 2 years of their offspring’s life. However, it is important to highlight that only one study objectively measured postpartum PA levels after a group-based PA intervention [40]. One of the three studies in the review that measured self-reported PA had a statistically significant effect on postpartum women’ PA at the 2-month post-test period, but no follow-up measures were conducted to test whether these changes were sustainable [42].

There are a range of possible reasons why Cramp and Brawley’s study [42] was able to positively change postpartum women’s self-reported PA, compared with the other studies in this review. First, the study focused only on delivering a group-based PA program (it was unifaceted in design), rather than targeting other behaviors, such as nutrition or parenting techniques. This meant that the outcomes, program content, and delivery, as well as outcome measurements were constructively aligned and clear to participants. The PA results in this systematic review are similar to the results in Gilinsky’s systematic review [24] which found a difference in effectiveness between postpartum PA interventions solely targeting physical activity and postpartum multifaceted interventions which targeted PA and other health behavior outcomes. Multifaceted postpartum programs which target a range of behaviors provide less PA opportunities and therefore had a lesser impact on PA behaviour. This suggests that the design of future PA interventions for postpartum women need to solely focus on providing PA opportunities that meet PA recommendations and should concentrate on measuring PA objectively to adequately capture changes in postpartum PA behaviors.

Second, it is possible the dose of the Cramp and Brawley’s study [42] led to the self-reported changes in PA. It was one of two studies [42, 46] included in this review that gave postpartum women an opportunity to participate in instructor-led group PA sessions (a combination of cardiovascular and muscle strengthening activities) for 60-min at least twice a week. This dose of PA assisted postpartum women in working towards the minimum requirement suggested by the PA guidelines of 150 min per week [9, 10] and led to the statistically significant improvements in self-reported PA [the other study [46] did not measure PA]. This is an important finding considering that the most recent recommendations delivered by the WHO PA guideline group [48] that highlights the need for future PA research with postpartum women to determine a dose of PA during this period that has an impact on PA and other health behavior outcomes. However, considering these important design factors, Cramp and Brawley’s study [42] was only rated a four in the assessment of study quality (see Table 3) and only measured PA using self-reported measures, so the findings of this study should be interpreted with caution. Thereby, there is a need to continue to evaluate the effects of unifaceted group-based PA programs in higher-quality randomized controlled trials, which use objective measures of PA and delivers a dose of PA that provides opportunities for postpartum women to meet minimum PA guidelines.

The type of PA utilised in group-based PA programs varied widely in the studies identified in this systematic review. Four of the studies focused on delivering cardiovascular and muscle strengthening activities for a period ranging from 40 to 60 min a session [42–44, 46] and the other two studies focused on delivering yoga and pram walking activities [40, 45]. The cardiovascular and muscle strengthening activities sessions have been highly recommended in recent PA guidelines, as available evidence from intervention trials combining both aerobic and muscle-strengthening PA show that postpartum women are more likely to attain PA guidelines and experience health enhancing benefits [49]. In addition, the cardiovascular and muscle strengthening sessions that were designed in the studies included in this review embedded pelvic floor strengthening activities. This is an important consideration as pelvic floor strengthening activities are often planned for and initiated in the immediate postpartum period, but current research suggests that this should be continued throughout the postpartum period, especially for those who do engage in moderate to vigorous PA [50]. However, due to the heterogeneity in terms of study design, outcomes, intervention design, sample size and outcome measurements among the studies, we cannot conclude which approach or type of activity had a larger effect on postpartum women’ PA levels. Further research is required to investigate the dose of cardiovascular and muscle strengthening sessions that is effective for improving outcomes, particularly for postpartum women [48].

Evidence suggests that interventions developed using a theory of behavior change (e.g., Social Cognitive Theory, Transtheoretical Model) and that target the hypothesized mediators of behavior change are more successful in changing behavior than atheoretical ones [51]. This premise is supported by a recent systematic review and meta-analysis that aimed to describe the associations between behavioral change strategies and changes in weight, diet, and PA in postpartum women [35]. Findings of the meta-analysis highlight that problem solving, goal setting, reviewing outcomes, receiving feedback and self-monitoring strategies were associated with larger changes in postpartum women’s health behaviors [35]. This is supported by this systematic review, with two of the three studies underpinned by a theoretical framework [40, 42, 44] significantly improving postpartum women’s self-reported PA [42], self-regulatory efficacy [42] and feelings of fatigue [40]. These studies embedded a range of behavior change strategies, including counselling sessions to develop self-regulatory skills for self-management of PA [42] and workbooks and pedometers to structure goal-setting, planning, and self-monitoring plans around PA [40]. Future research focusing on group-based PA programs for postpartum women should be underpinned by an appropriate theoretical framework that embeds a range of behaviour change strategies.

Overall, the group-based PA interventions included in this review were somewhat successful in changing or increasing postpartum women’ psychological wellbeing. There is growing evidence that regular moderate-intensity PA during the postpartum period may assist in attenuating depressive symptoms and improving mental health and wellbeing [52, 53]. All but one of the studies included in this review, measured postpartum women’s mental health and wellbeing (e.g., psychological wellbeing, perceived stress, mood, coping, fatigue – see Table 2), with one of the studies having psychological wellbeing as the primary outcome [46]. The study that had psychological wellbeing as its primary outcome, was the only study to report positive and statistically significant changes in psychological wellbeing [46]. This is likely, as the postpartum women in this study were engaged in group-based PA sessions three times per week, which contributes to the growing evidence that regular participation in moderate-intensity PA has psychological benefits [52]. However, although qualitative or process data was not collected from the participants in this study [46], factors such as enjoyment, mastery of skills, attainment of goals, autonomous motivation, choice, social interaction, and a sense of belonging [54] are also likely to influence the relationship between participation in a PA intervention and impact on psychological wellbeing and mental health. Another consideration, due to building evidence around the role of social interaction in the PA and mental health relationship [55] further research is needed to examine whether PA with other postpartum women is more beneficial than PA alone. Hence, future research focusing on postpartum women and participation in PA, should consider qualitative data to capture more information about what, when, where, who, and how. This will provide necessary data on intervention characteristics needed for developing group-based PA programs for postpartum women.

### Strengths and limitations of the study

To our knowledge, this is the only systematic review of international literature aimed at synthesizing data regarding group-based PA interventions and impact on postpartum women’s PA or other health behavior outcomes. Many important findings have been reported leading to the recommendations for future research. However, this systematic review and narrative synthesis also has its limitations. First, the review only included articles written in English. The six studies included were implemented in six different countries, which limits the evidence that may be accrued for each population of postpartum women in each country. Despite the variety, all the study’s interventions were conducted in high income (e.g., OECD) countries. Therefore, findings from this review should be limited to informing decision marking of researchers and other stakeholders in those of similar nations.

Despite the growing number of PA interventions for postpartum women [35], very few have focused on designing group-based PA strategies and activities for a healthy population of postpartum women. As a result, it was not possible to conduct a meta-analysis on the six included studies and meaningfully test the potential effects of confounds. In addition, studies in this review included a variety of intervention outcomes, structures, and measurement tools to assess PA (e.g., accelerometers and self-reported measures), and health behavior outcomes. While the measures reported in each study used validated and reliable measures, different measures may produce different estimates, especially when measuring PA, and it is possible that different measures could be more or less sensitive [56]. It is also important to acknowledge that as the search strategy was conducted by searching for terms in the titles and abstracts fields. As such, poorly reported studies or studies that had physical activity as a secondary outcome may not have been included in this systematic review. As additional studies emerge, it will be important to update this review, conduct a meta-analysis and account for potential confounding factors.

## Conclusion

In summary, we have identified the few studies that have designed and developed group-based PA programs for postpartum women. Presently, group-based PA programs have not been successful at significantly increasing objective measures of PA and have had limited success at significantly increasing self-reported PA, as well as psychological wellbeing and fatigue. To strengthen the evidence-base for group-based PA programs with postpartum women there is an on-going need for more rigorous randomised controlled trials of appropriate length (at least 3 months in duration) with an adequate dose of group-based PA sessions per week that meet PA guidelines, and utilise objective measures of PA. In addition, future PA interventions for this population should include, at the very least, fidelity and process data to capture the characteristics or design features that appeal most to postpartum women. This systematic review and narrative synthesis have highlighted the need for continuity of outcome variables and instruments of measurement to be used across studies. Meta-analytic work in this space will be unreliable until these issues are addressed, and less heterogeneity exists between studies.

## Supplementary Information

**Additional file 1**. S1: Search strategy.

## Data Availability

The databases used in the study were all open access and included the following electronic databases (MEDLINE, CINAHL, EMBASE and PsychInfo). The datasets used and/or analysed during the current study are available from the corresponding author on reasonable request.

## Abbreviation

PA: Physical activity
