# Supplementary material for: Group-based physical activity interventions for postpartum women with children aged 0–5 years old: a systematic review of randomized controlled trials
Source: BMC Womens Health. 2021 Dec 28;21:435. doi: 10.1186/s12905-021-01581-1 (PMC8714424; doi:10.1186/s12905-021-01581-1)
Supplement: Supplementary file 1 — Additional file 1. S1: Search strategy. [file 12905_2021_1581_MOESM1_ESM.docx]

***S1: Search strategy***

**Medline**

(("Postpartum"[Mesh] OR postnatal OR post-natal OR puerperium OR postpartal OR post-partal OR lactating OR lactation OR “nursing women” OR breastfeeding OR breastfeeding OR “after birth” OR “following pregnancy” OR postpregnancy OR “post pregnancy” OR “following childbirth” OR “after delivery” OR “post childbirth”)) AND (("Physical Activity"[Mesh] OR exercis* OR fitness)) AND ((group [MESH]* OR team OR leadership OR facilitat*)) AND (“randomized controlled trial*” OR “Clinical Trial*” OR “randomised controlled trial*” OR RCT OR “random allocation” OR “randomly allocated” OR “allocated randomly” OR “control” OR “trial” OR “evaluat*” OR “quasi- exper*” OR “cluster” OR “intervention*”). Filters for: Humans, English, Peer-reviewed journals only, Date 2000-Oct 31 2020.

**PsycINFO**

Title and Abstract: Postpartum OR postnatal OR post-natal OR puerperium OR postpartal OR post-partal OR lactating OR lactation OR “nursing women” OR breastfeeding OR breastfeeding OR “after birth” OR “following pregnancy” OR postpregnancy OR “post pregnancy” OR “following childbirth” OR “after delivery” OR “post childbirth”)) AND Title and Abstract "Physical Activity" OR exercis* OR fitness AND Title and Abstract group* OR team OR leadership OR facilitat* AND Title and Abstract “randomized controlled trial*” OR “Clinical Trial*” OR “randomised controlled trial*” OR RCT OR “random allocation” OR “randomly allocated” OR “allocated randomly” OR “control” OR “trial” OR “evaluat*” OR “quasi- exper*” OR “cluster” OR “intervention*” AND Peer-Reviewed Journals only AND 2000- Oct 2020.

**CINAHL**

Title and Abstract: Postpartum OR Title and Abstract postnatal OR Title and Abstract post-natal OR Title and Abstract puerperium OR Title and Abstract postpartal OR Title and Abstract post-partal OR Title and Abstract lactating OR Title and Abstract lactation OR Title and Abstract “nursing women” OR Title and Abstract breastfeeding OR Title and Abstract breastfeeding OR Title and Abstract “after birth” OR Title and Abstract “following pregnancy” OR Title and Abstract postpregnancy OR Title and Abstract “post pregnancy” OR Title and Abstract “following childbirth” OR Title and Abstract “after delivery” OR Title and Abstract “post childbirth”)) AND Title and Abstract "Physical Activity" OR Title and Abstract exercis* OR Title and Abstract fitness AND Title and Abstract group* OR Title and Abstract team OR Title and Abstract leadership OR Title and Abstract facilitat* AND Title and Abstract “randomized controlled trial*” OR Title and Abstract “Clinical Trial*” OR Title and Abstract “randomised controlled trial*” OR Title and Abstract RCT OR Title and Abstract “random allocation” OR Title and Abstract “randomly allocated” OR Title and Abstract “allocated randomly” OR Title and Abstract “control” OR Title and Abstract “trial” OR Title and Abstract “evaluat*” OR Title and Abstract “quasi- exper*” OR Title and Abstract “cluster” OR Title and Abstract “intervention*” AND Peer-Reviewed Journals only AND 2000-2020.

**EMBASE**

(("Postpartum"[EMTREE] OR postnatal OR post-natal OR puerperium OR postpartal OR post-partal OR lactating OR lactation OR “nursing women” OR breastfeeding OR breastfeeding OR “after birth” OR “following pregnancy” OR postpregnancy OR “post pregnancy” OR “following childbirth” OR “after delivery” OR “post childbirth”)) AND (("Physical Activity"[EMTREE] OR exercis* OR fitness)) AND ((group [EMTREE]* OR team OR leadership OR facilitat*)) AND (“randomized controlled trial*” OR “Clinical Trial*” OR “randomised controlled trial*” OR RCT OR “random allocation” OR “randomly allocated” OR “allocated randomly” OR “control” OR “trial” OR “evaluat*” OR “quasi- exper*” OR “cluster” OR “intervention*”). Filters for: Humans, English, Peer-reviewed journals only, Date 2000-Oct 31 2020.
